# The FRAME: an expanded framework for reporting adaptations and modifications to evidence-based interventions

**DOI:** 10.1186/s13012-019-0898-y

**Published:** 2019-06-06

**Authors:** Shannon Wiltsey Stirman, Ana A. Baumann, Christopher J. Miller

**Affiliations:** 1 National Center for PTSD and Stanford University, 795 Willow Road NC-PTSD, Menlo Park, CA 94025 USA; 2 Washington University in St. Louis, One Brookings Drive, St. Louis, MO 63130 USA; 3 Center for Healthcare Organization and Implementation Research (CHOIR),VA Boston Healthcare System, Boston, MA 02130 USA; 4 Harvard Medical School Department of Psychiatry, Boston, MA 02115 USA

**Keywords:** Modification, Adaptation, Cultural adaptation, Implementation outcomes

## Abstract

**Background:**

This paper describes the process and results of a refinement of a framework to characterize modifications to interventions. The original version did not fully capture several aspects of modification and adaptation that may be important to document and report. Additionally, the earlier framework did not include a way to differentiate cultural adaptation from adaptations made for other reasons. Reporting additional elements will allow for a more precise understanding of modifications, the process of modifying or adapting, and the relationship between different forms of modification and subsequent health and implementation outcomes.

**Discussion:**

We employed a multifaceted approach to develop the updated FRAME involving coding documents identified through a literature review, rapid coding of qualitative interviews, and a refinement process informed by multiple stakeholders. The updated FRAME expands upon Stirman et al.’s original framework by adding components of modification to report: (1) when and how in the implementation process the modification was made, (2) whether the modification was planned/proactive (i.e., an adaptation) or unplanned/reactive, (3) who determined that the modification should be made, (4) what is modified, (5) at what level of delivery the modification is made, (6) type or nature of context or content-level modifications, (7) the extent to which the modification is fidelity-consistent, and (8) the reasons for the modification, including (a) the intent or goal of the modification (e.g., to reduce costs) and (b) contextual factors that influenced the decision. Methods of using the framework to assess modifications are outlined, along with their strengths and weaknesses, and considerations for research to validate these measurement strategies.

**Conclusion:**

The updated FRAME includes consideration of when and how modifications occurred, whether it was planned or unplanned, relationship to fidelity, and reasons and goals for modification. This tool that can be used to support research on the timing, nature, goals and reasons for, and impact of modifications to evidence-based interventions.

## Background

Adaptation, a key concept in implementation, has been defined as a process of thoughtful and deliberate alteration to the design or delivery of an intervention, with the goal of improving its fit or effectiveness in a given context [1, 2]. It is a form of modification, which is a broader concept that encompasses any changes made to interventions, whether deliberately and proactively (adaptation), or in reaction to unanticipated challenges that arise in a given session or context [3, 4]. The process, nature, and outcomes of modifications to evidence-based programs/practices (EBPs) have often not been well documented, despite considerable recent interest in the field of implementation science [5, 6]. Consequently, modification has historically not been fully evaluated or understood.

Understanding what, how, and when modifications occur is a vital aspect of implementation science because the process of implementing EBPs is dynamic [7]. Modifications may occur for a variety of purposes and with differing implications. Some may enhance outcomes, particularly if they more closely align the intervention with the needs of the specific population in a particular system or context. In fact, modifications that focus on increasing the fit of the EBPs with the target population can lead to improved engagement, acceptability, and clinical outcomes, particularly when working with minority populations [8–10]. However, modifications that remove key elements of an intervention, or fail to align with population needs, may be less effective [7, 11–14]. Inconsistent reporting has resulted in uncertainty regarding modification’s impact on health and the types of modifications that can maximize implementation success [15, 16].

Without understanding forms of modification that occur, the systematic evaluation of processes and strategies that lead to more and less successful implementation may be hindered [2, 17–19]. To facilitate a more nuanced consideration of modifications and to work toward identifying forms of modifications that may enhance specific interventions vs. those that may reduce effectiveness [16], Stirman and colleagues previously developed a Framework for Modification and Adaptations [16]. This framework characterized different forms of modifications to interventions, and later work differentiated fidelity-consistent from fidelity-inconsistent modification [1, 2, 16]. More recently, other research groups have used the framework to characterize modifications to various healthcare interventions and prevention programs [20–23]. Some key aspects of the 2013 framework were shown to have high clarity (rater agreement) and acceptable to high coverage (percentage of identified adaptations that could be classified using the taxonomy; [24]).

Despite its value in distinguishing and categorizing different forms of modifications, the original framework did not capture other considerations that may be important to document. Because it was originally developed largely to identify forms of modification rather than to fully document the process itself, it did not include potential reasons for modifications, which can range from improving individual or contextual fit (e.g., [20, 21, 25, 26]) to addressing systemic constraints. In 2017, Baumann et al. consulted the implementation literature and the literature on social determinants of health and engaged in a consensus process to expand the framework to include possible reasons for adaptation [15], a process that differed from the original process of framework development. They recommended additional work to refine the resulting framework.

As we planned a process to refine the framework further, we identified additional limitations and opportunities for expansion. For example, while the 2013 and 2017 frameworks [15, 16] laid out distinct forms of modification that could be made to the content or mode of delivery, they did not specify when those modifications were designed to address important differences between the original population and the stakeholders in the current implementation effort (i.e., cultural adaptations). The frameworks also did not specify whether modifications were planned (i.e., adaptations) or unplanned (e.g., [2, 4, 27]), or allow investigators to consider modifications in conjunction with fidelity, a related but distinct implementation outcome [1]. Further, we recognized that other aspects of reporting that may be important for improving understanding of modification and its impact, such as when in the implementation process the modification was made, were also not included [28–31]. To address the aforementioned issues, our goal was to develop a refined framework that expanded the original framework to facilitate documentation of additional aspects of the implementation process.

### Process for refining the framework

We employed a pragmatic, multifaceted approach, detailed in Table 1, to develop a more comprehensive strategy for characterizing adaptation design and process. This approach included multiple sources of data that better aligned with our goals than approaches such as scoping or systematic reviews [32]. We chose this pragmatic approach because several systematic reviews of adaptation have been published in recent years, and we recognized that such reviews might not capture aspects of modification and adaptation that had not been adequately documented.

**Table 1:** Process of refining the framework

Steps	Process/operationalization
1. Identify goal and scope	Goals: Identify reasons for adaptation not presented in Baumann et al. [15]; determine other aspects of the modification or adaptation process that should be documented
2. Identify relevant literature	Searched the literature for systematic reviews and adaptation frameworks (additional details about search terms and processes available from first author) employed a snowballing process to sample underlying source literature for review (SWS)
3. Identify information about adaptation that was not captured in the previous framework	1. Identify descriptions of the process and reasons for adaptation in the published literature 2. Compare to the existing framework 3. Extract novel (a) descriptions and categorizations of modifications and adaptations, (b) reasons for modification or adaptation, (c) recommendations for adaptation, (d) descriptions of the process of adaptations, and (e) discussions of limitations of the existing frameworks and adaptation literature (SWS) 4. Added novel descriptions and information to a spreadsheet, employing a stop rule (e.g., when no additional information is extracted in the subsequent 10 articles)
5. Rapid coding of 55 interviews	Reviewed memos and notes generated by two trained research assistants who applied the 2017 framework to questions about the adaptation process. Extracted summaries about aspects of adaptation not included in the framework and added to a spreadsheet (SWS)
6. Check extraction results for completeness	Reviewed a subset of articles and interview responses to ensure that extraction was complete (CM, AB), arrived at consensus using a stop rule (e.g., when no additional information is extracted in the subsequent 10 articles)
7. Classify resulting items to create a complete list of possible reasons for adaptation and specify other aspects of the adaptation process to be documented	Reviewed the items extracted from the literature and from interviews to categorize reasons for adaptation. Compare the information from the two data sources and finalize broad categories. Collapse and organize similar subcategories (SWS, AB, CM; by consensus).
8. Integrate stakeholder feedback	Presented the revised framework, along with the rationale and methodology, to three different groups of stakeholders (implementation researchers, implementation project leaders, practitioners, and intervention developers) at seven different meetings with an explicit request for feedback. Suggestions regarding additions, refinements, and clarifications were recorded, discussed, and added to the framework by team consensus (SWS, AB, CM)
9. Piloted framework	Coded articles with a predetermined stop rule (planned stop when no additional information that was not covered in FRAME was identified after 10 articles). Ten articles were coded with no new information identified

### Review of the literature

We first searched the literature and identified existing frameworks, systematic reviews, and discussions of adaptations of public health and behavioral health interventions that had been published since 2013 [1, 6, 29, 33]. We also drew on systematic reviews of intervention modifications and adaptations, existing adaptation frameworks, and reviews on cultural adaptation [3–5, 15, 17, 20, 21, 33, 34]. We employed a snowballing approach to examine a total of 170 individual articles in the literature that described adaptations to interventions. After reviewing all articles from three systematic reviews [1, 17, 29], we implemented a “stop rule” such that if no new aspects of adaptation were identified after examining 10 original sources from each additional systematic review, we ceased reviewing individual articles. Additionally, we examined two widely cited frameworks of implementation that listed potential determinants [30, 35] and a framework of social determinants of health [36, 37] to further refine the reasons for adaptation. After steps 1–8 (Table 1) were complete, we piloted the resulting framework by coding a sample of articles and interview responses to ensure that no additional elements of adaptation were identified, once again implementing the 10-article “stop rule”.

### Reviewing qualitative data

To complement our literature review, we conducted a rapid coding process with a set of responses to questions about adaptation. We generated the responses from interviews with 55 mental health providers and administrators that detailed processes and reasons for adaptation of a psychosocial intervention (see Table 1). These interviews were conducted through studies on sustainability and adaptation [2, 38].

### Data consolidation

Using the information generated through the two data sources, we categorized both reasons for adaptation and aspects of the adaptation process not captured in the previous framework. We then collapsed similar subcategories and organized them by consensus among the three authors.

### Framework refinement

Finally, to increase the likelihood that the updated framework would document aspects of modification and adaptation that were important to stakeholders, we presented a draft of the framework to several groups of stakeholders and solicited suggestions regarding additions, refinements, and clarifications.

### Overview of the resulting framework and reporting recommendations

**Fig. 1:**
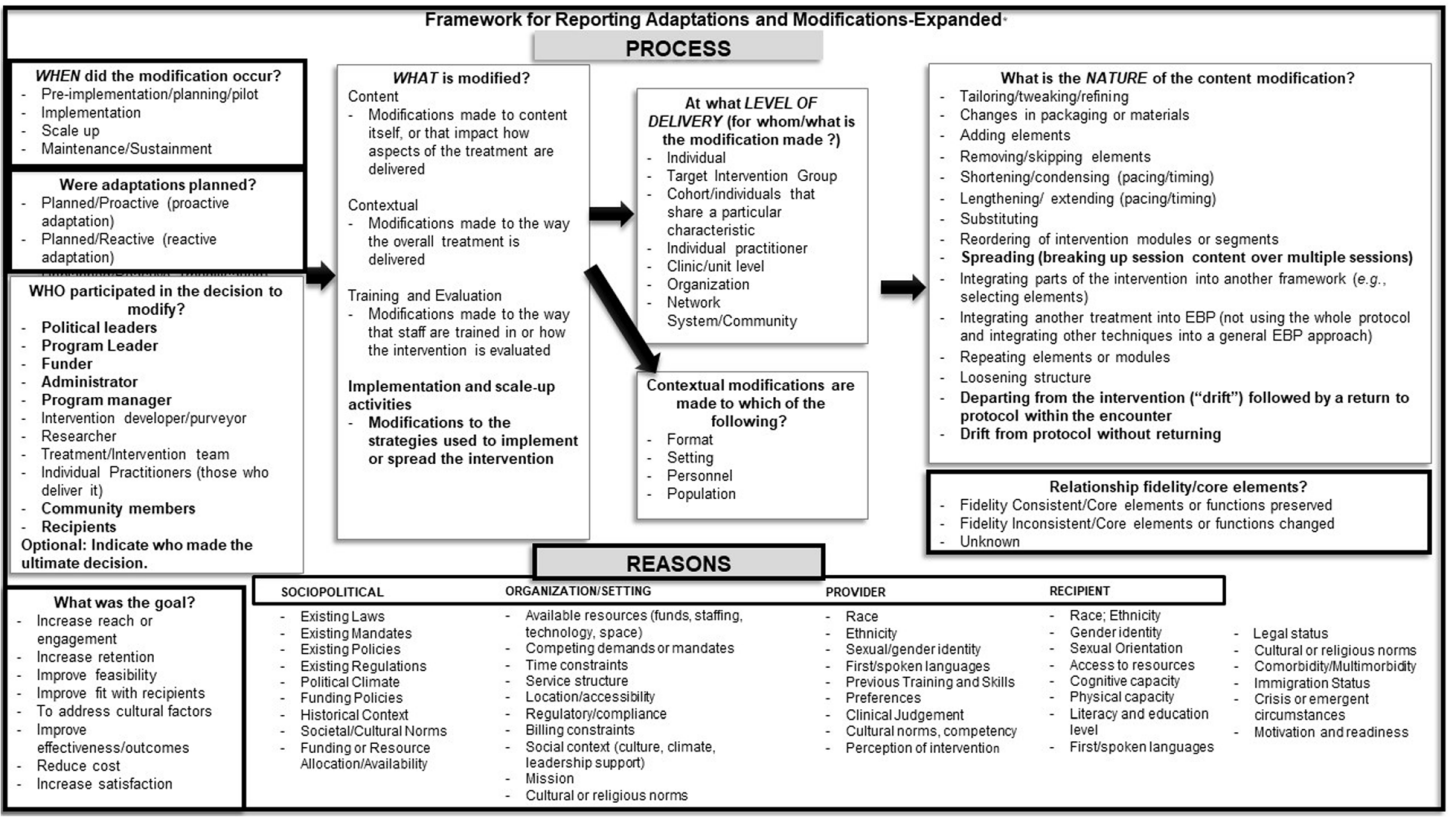
The Framework for Reporting Adaptations and Modifications-Expanded (FRAME). New elements are outlined in black lines, while the original aspects of the 2013 framework are outlined in gray. Additions and refinements within categories included in the 2013 framework are italicized. Recommended elements of reporting were as follows: (1) when and how in the implementation process the modification was made, (2) whether the modification was planned/proactive (i.e., an adaptation) or unplanned/reactive, (3) who determined that the modification should be made, (4) what is modified, (5) at what level of delivery the modification is made, (6) type or nature of context or content-level modifications, (7) the extent to which the modification is fidelity-consistent, and (8) the reasons for the modification, including (a) the intent or goal of the modification (e.g., cultural adaptations, to reduce costs, etc.) and (b) contextual factors that influenced the decision. Adapted from (Baumann A, Cabassa LJ & Stirman SW, 2017; Stirman SW, Miller CJ, Toder K & Calloway A, 2013)

Our approach yielded several additions and refinements to the original framework, which are indicated in bold in Fig. 1. Just as Proctor and colleagues [39] advocate for a multifaceted approach to reporting implementation strategies, our framework is intended to facilitate comprehensive documentation of modifications. Our Framework for Reporting Adaptations and Modifications-Enhanced (FRAME) includes the following eight aspects: (1) when and how in the implementation process the modification was made, (2) whether the modification was planned/proactive or unplanned/reactive, (3) who determined that the modification should be made, (4) what is modified, (5) at what level of delivery the modification is made, (6) type or nature of context or content-level modifications, (7) the extent to which the modification is fidelity-consistent, and (8) the reasons for the modification, including (a) the intent or goal of the modification (e.g., improve fit, adapt to a different culture, reduce costs, etc.) and (b) contextual factors that influenced the decision. Below, we describe in further detail aspects of the FRAME, with attention to aspects not included in the original framework.

### When and how in the implementation process the modification was made

Timing, not included in the original framework, is important to measure as modification can occur in any phase of the long-term implementation process: pre-implementation activities, an implementation phase, and scale-up and sustainment [40–42]. During the planning or pre-implementation phase, there are opportunities to anticipate changes and discover adaptations that need to be made through a pilot phase. Despite these efforts, new challenges, constraints, or potential enhancements may come to light during the implementation phase. During scale-up or scale-out, other changes may be necessary, particularly for contexts where the intervention is likely to reach populations that may differ from the population that received the intervention during implementation [43]. Additionally, during sustainment, changes in the system or population served by that system may also necessitate changes [7, 44, 45].

### Whether the modification was planned/proactive or unplanned/reactive

Reporting on when and how decisions to modify interventions are made will allow investigation of whether planned adaptations are different in nature or in outcomes than those that are improvised during implementation. To avoid unplanned or reactive modifications that are inconsistent with an intervention’s goals, research base, or theory, investigators have advocated a process of planned adaptation [46], ideally as early as possible in the implementation process. However, in practice, many modifications are made less systematically [4]. Reactive modifications have been defined as those that “occur during the course of program implementation, often due to unanticipated obstacles” [47]. These modifications often occur in an impromptu manner, in reaction to constraints or challenges that are encountered [3], and may or may not be aligned with the elements of the intervention that make it effective [2, 4].

Adaptations are typically made proactively through a planning process that identifies ways to maximize fit and implementation success while minimizing disruption of the intervention [10]. However, due to the reactive nature of other modifications, we added a specifier to the framework to capture whether modifications were planned prior to delivery (i.e., an adaptation), or unplanned and made in response to an unanticipated challenge. It is important to note that iterative changes are not necessarily reactive—iteration can accommodate unanticipated challenges. For example, a modification made during the “Act” portion of a “Plan-do-study-act” cycle would not be considered reactive, because it was determined through a systematic process rather than through improvisation.

### Who determined the modification

The driver of change, and how participatory the decision was, may be important predictors of whether the changes have the desired impact. The FRAME can be used to list all who play a role in the decision, but in some contexts, it may be important to specify who makes the ultimate decision, as this detail might affect whether and how widespread the modification may occur. It may also be closely linked to reasons for modification. For example, administrators’ decisions to modify an EBP may be related to restrictions in sourcing or contracting while policymakers’ decisions may be in response to political factors or funding availability. Modifications made by individual providers may respond to recipient-level needs or local constraints that may not be visible to policymakers.

### What is modified

The original 2013 framework [16] focused largely on characterizing what types of changes were made to facilitate understanding of which changes are associated with implementation success and recipient-level outcomes. Much of this component of the framework remains unchanged, although we added implementation and scale-up activities to reflect that these processes may differ across contexts. If little about the intervention were changed, but the implementation strategies differed significantly across otherwise similar contexts, then differences in outcomes may be attributable to differences in how the intervention was implemented.

### At what level of delivery the modification is made

Codes for the level of delivery also remain mostly unchanged from the 2013 framework. Reporting the level at which modifications occur has implications for understanding whether and under what circumstances implication success or effectiveness are associated with making individual-level modifications, and when modifications may need to be applied more broadly. The FRAME now differentiates the entire target group (e.g., women who are at risk of developing diabetes) and individual sub-groups (e.g., new mothers who are at risk of developing diabetes), as modifications may be made for broad or specific groups depending on the circumstances.

### Type or nature content-level modifications

Although many items in this section are unchanged from the original framework, items were added to reflect a larger variety of modifications identified through the literature, ongoing observational work (c.f., [1, 3, 20, 21, 23, 48]), and stakeholder interviews. For example, it may be important to understand whether drift occurs for a relatively brief period of time before returning to a protocol or whether it occurs for the duration of a meeting or session, without a return to planned content. Additionally, spreading out psychosocial or educational content intended for a single meeting or session over multiple sessions was added, as this may occur when an individual requires more time to understand content or when unforeseen or emergent issues need to be addressed during a given session.

### The relationship to fidelity

In previous work, we have made a distinction between fidelity-consistent and fidelity-inconsistent modifications. Fidelity-consistent modifications are defined as those that preserve core elements of a treatment that are needed for the intervention to be effective [49]. In contrast, fidelity-inconsistent modifications are those that alter the intervention in a manner that fails to preserve its core elements. The identification of fidelity-consistent and fidelity-inconsistent modifications can be made in consultation with the existing literature, input from the treatment developer, and any available evaluation data. Others have suggested that the function of intervention elements be prioritized over the form, such that what is core to the intervention is conceptualized as one of several possible activities or materials that accomplish a core intervention function (e.g., education, skill building, connecting to supports or resources) [50].

When implementing an intervention with a population that differs in important ways from the populations with which the intervention has been tested and implemented, new understanding of which elements are core vs. peripheral may emerge. For example, a component intended to improve basic health literacy may be essential for one population, but less necessary for a population that has a solid foundation of specific health information. Moreover, it may not be known whether some modifications, such as preserving the original spacing of sessions or activities, would have an impact on outcomes, and there may not be theory to inform decisions about such modifications either way. We have therefore added an “unknown” code that can be used when there is no theory or evidence to inform a decision about whether an element is core vs. peripheral.

### The rationale for the modifications made

The additions related to the rationale for modifications represent the most significant enhancements to the original framework. Capturing the rationale for a given modification may be crucial in determining links to key implementation or health outcomes [20]. For example, modifications made to cut costs may have a very different impact than those made to improve fit or engagement. Thus, we specify the *goal for modification*—to improve feasibility or acceptability, to increase reach or engagement, to improve fit (note that cultural modifications intended to improve fit are assigned a subcategory under this goal), to reduce costs, to improve clinical outcomes, or to align the intervention with cultural values, norms, or priorities.

To identify *reasons to modify* EBPs for FRAME, we referred to existing reviews and original literature that enumerated reasons for specific projects and implementation frameworks that specified potential determinants at different levels. We specifically added cultural modifications within the FRAME due to the importance of cultural modifications in implementation science. Attention to cultural factors is important in investigating modifications to EBPs because of its potential consequences in terms of healthcare disparities [15]. Cultural adaptation can be defined as the systematic modification of an intervention to “consider language, culture, and context in such a way that it is compatible with the client’s cultural patterns, meanings, and values” [35]. Focusing on culture allows us to expand the characterization of content modifications (e.g., whether content is added, removed, or tailored) and context-level modifications (e.g., whether personnel, training, or education are modified) to explicitly address cultural patterns or values at the client, provider, or sociopolitical level. Designation of cultural or religious norms at the organizational level distinguish adaptations made to distinguish this form of culture from the construct of organizational culture used in the implementation and organization literature to signify “the way things are done in an organization” [51]. At times, cultural or religious beliefs may contribute to organizational culture or policies, but the constructs do not fully overlap.

By explicitly defining cultural aspects and determinants that affect inequities in care delivery, we hope to identify the types of modifications made to address cultural aspects of the populations included in our studies. Thus, we also examine, more broadly, factors at the recipient, provider, organizational, and socio-political levels that are examples of important components that affect the modifications of the EBPs.

*Socio-political factors* may also be important determinants of modifications to EBPs captured in the FRAME. For example, socio-historical factors such as stigma attached to receiving mental health treatment may require modifications so that interventions are instead delivered by spiritual leaders or peers, or in settings that are more comfortable for members of a community. Existing laws and policies can impact whether aspects of an intervention are removed due to constraints (e.g., telephone check-ins may be removed if they cannot be reimbursed; licensure restrictions for telehealth across state lines) and may also result in adaptations to personnel or setting.

*At the organizational level*, we found that factors that may lead to modifications overlap somewhat with those found in existing determinant frameworks (e.g., [41, 52]). For example, staffing shortages may suggest a variety of context modifications. This may include delivery by different personnel (e.g., providers from a different discipline) or changes to the format or timing of delivery. Such staffing shortages may also affect training or evaluation of the intervention. For example, implementation may require streamlining training sessions, adapting them to fit with providers’ previous training, or spreading them over several weeks to accommodate busy clinic schedules.

Other practical constraints play a role in decisions to adapt or modify interventions [2, 15, 19, 23]. Space shortages may indicate the need for context modifications (e.g., changing from group to individual delivery). In contexts without easily accessed health centers, community-, home-, or telehealth-based delivery formats, there may be contextual adaptations to address demand or need. Limitations to available technology can have far-reaching implications as well as it may require removal or adaptation of aspects of the original intervention that can be delivered in under-resourced settings. Time constraints may lead to removing elements or compressing the intervention.

Aspects of organizational/setting (including local community if not delivered in a healthcare setting) culture may also impact how interventions are delivered. Competing demands, de-prioritization of an intervention, or high rates of turnover may lead to changes in who delivers the intervention, how many sessions or elements are provided, and whether and how training is provided. Regulatory or compliance issues or legal concerns may lead to certain aspects of an intervention not being delivered (e.g., limits to the types of physical activity or activities that may occur off-premises). An organizational culture that has long espoused a different theory or intervention may leave providers wary of new practices and lead to integration of elements of interventions into existing practices rather than de-implementation of preferred practices [53]. In sum, organizational/setting factors—including organizational culture and available resources—may necessitate a variety of modifications even to interventions with a strong evidence base in specific contexts.

*At the provider level*, there has been discussion around the positive or negative consequences of modifying interventions to fit with provider preferences or to improve the interaction between providers and their clients [1]. Providers of psychosocial interventions frequently modify interventions for a variety of reasons, including perceived client preferences, providers’ preferences or self-efficacy [54], and efforts to maintain a good therapeutic alliance [55–58]. Factors such as provider gender and cultural beliefs may also impact decisions about delivery of the intervention. Additionally, some provider factors, such as previous training and experience, may lead to changes to training and evaluation.

*Recipient level* factors are also identified in Fig. 1. Each identified factor may contribute to a need for modification to promote optimal levels of engagement and outcomes at different levels. For example, limited transportation might make face to face meetings in a clinic difficult, which could lead to modifications in the format (e.g., telephone or internet-based) or setting (home-based). On the other hand, low literacy might make full engagement in an intervention that relies on written materials difficult and require tailoring to deliver content to clients in other formats. Other aspects, such as cultural norms, legal status, or physical capacity, may necessitate the removal or alteration of some elements of the EBP.

### Limitations

Although our approach to refining the framework is unique in its use of multiple data sources and stakeholder input, some limitations are important to describe. We did not employ a systematic review or a traditional thematic analysis because these approaches did not fully align with the current project goals. While it is possible that additional items would have been identified through these processes, our use of a “stop rule”, stakeholder feedback, and coding of subsequent articles using the FRAME after consolidating information from all of our data sources suggested that the framework was sufficiently comprehensive.

Furthermore, stakeholders identified a need to balance comprehensiveness with feasibility and pragmatism in documentation and reporting. The feasibility of using a comprehensive framework is likely to differ across research and applied settings. We must also acknowledge that comprehensively cataloging modifications to EBPs may be difficult in some contexts even with a well-developed framework like the FRAME. For example, if an intervention has not been exhaustively described and tested—as is frequently the case in healthcare—then it may be impossible to reliably detect adaptations and their associated consequences. In these cases, the FRAME will only be as useful as the data informing its application. Evaluation of different approaches to using the FRAME for documentation is needed to inform efforts to achieve the appropriate balance.

### Recommendations and future directions in reporting

Several strategies for reporting adaptations and modifications that may be applicable to the FRAME have been developed and described in the literature. In this section, we discuss the advantages and drawbacks to these strategies.

#### Observation

To identify modifications that are made during routine treatment delivery, Stirman and colleagues developed the Modification and Adaptation Checklist (MAC; [59]), an observational coding system intended to be used in conjunction with fidelity assessments. Observation, the “gold standard” for fidelity coding, may be useful when providers may not realize they are making content modifications or when they have difficulty recalling, identifying, or describing which modifications they made. It may be particularly useful in contexts in which providers may be reluctant to report modifications such as drift or removing key intervention elements. However, observation is not feasible in many contexts as it is labor intensive and would require familiarity with the FRAME and the intervention. Intermittent observation, although more feasible, might lead investigators to miss certain forms of modification, such as extending a protocol or repeating material in a session or encounter that is not observed [1]. Furthermore, without additional information from stakeholders, the rationale for making a modification cannot be confirmed.

#### Provider or key informant self-report

A self-report version of the MAC includes both content-level modifications and brief questions about format, level of delivery, and reasons for modification. The reasons for modification are less detailed than those included in the FRAME. However, space for details about the specific contextual factors that were considered or that led to modification can allow for free responses that can be coded. Self-report may be more feasible when frequent assessment is required, although it may entail greater burden on providers than participating in a one-time interview or having encounters observed. Providers may over-report some forms of modification (c.f., [56]), while underreporting others. In fact, at times, providers may not recognize that they are modifying the interventions or whether adaptations are fidelity-consistent or fidelity-inconsistent. Core elements and functions of the intervention may not be fully established, making it challenging to report these aspects. These challenges may be heightened when modifications are not tracked in real-time, and reporting may be subject to recall bias, or when training has not been sufficient to promote awareness of fidelity. Additionally, incentives and contingencies may be present that impact reporting of modification and fidelity to an intervention.

Despite these limitations, self-report measures may be the most feasible strategy for real-time reporting of modifications that occur during implementation and sustainment phases. However, many of the context modifications that are reported can be validated through other forms of documentation. It remains to be determined whether self-report and observer ratings agree, and the optimal frequency for self-report. Additionally, it is unclear whether self-reports are more informative and accurate as global self-assessments (e.g., inquiring which interventions a provider has made over a given period of time) or for a single encounter or time point (e.g., focusing assessments on which intervention a provider used for a single encounter). Recent and ongoing research are attempting to address these types of questions [22, 38].

#### Interviews

Interviews may provide richer data than provider checklists or observation of single encounters, although they may be subject to similar biases as self-report. They allow an understanding of who made the ultimate decision to modify, the level of delivery, reasons for adaptation, and contextual factors that were considered. We developed a codebook for our original framework [16] to allow investigators to operationalize and identify modifications that were made to interventions during the implementation process. It was originally used for coding articles in the literature [16] and interviews with community-based clinicians [56] and has since been used in a variety of contexts [20–23]. Interview guides based on an expanded codebook can be used to facilitate understanding of the eight FRAME elements. For example, Rabin and colleagues recently described a measurement system that expanded the Stirman et al. 2013 framework to include RE-AIM concepts, framed as *Who*, *How*, *When*, *What*, and *Why*? [20]. A potential drawback to interviews is that they may not be feasible to administer frequently due to the time required for interviews and coding.

Differing forms of assessment will need to be compared to assess accuracy and reliability, and factors such as burden on stakeholders and research participants will need to be considered in determining the best assessment strategy for a given project. For some interventions, modifications may be most reliably identified through self-report checklists (with sufficient descriptions of each adaptation to facilitate reliable reporting) that are completed soon after the intervention is delivered, while others may be best identified through detailed interviews with stakeholders. Triangulation of strategies may be necessary when modifications are not easily observed and to better assess reasons for modification. For example, Rabin et al. [20] used observational data in addition to interviews to construct intervention process maps and identify additional contextual factors that may be relevant to adaptation.

### Future directions and research agenda

#### Measurement and reporting

While attention to modification has greatly increased in the past decade, the science of measurement and reporting remains nascent. Strategies for reporting and measurement have not yet been empirically compared, nor have psychometric properties of self-reports been examined. When used for research, detailed versions of a FRAME measure can facilitate comprehensive reporting and analyses. For the most precise coding, the FRAME figure itself could be used for each separate modification that was identified, with the reporter or interviewer circling the appropriate selection in each of the sections. However, elements that do not apply in a given context could be removed to streamline reporting and evaluation in routine care contexts. As with sustainability [44], it is unlikely that assessment of modification at a single time point will reflect the dynamic process of implementation [7]. Assessment at multiple time points will provide a richer understanding of why, how, and with what impact modification occurs in complex systems.

#### Linking and understanding modifications and outcomes

Ultimately, the FRAME is intended to facilitate understanding of associations between the process, types, and reasons that interventions are modified and key outcomes. Over time, such research may identify which aspects of the FRAME are particularly important to attend to when planning and documenting adaptations. Key outcomes to consider include increased, decreased, or unchanged levels of reach, diagnostic outcomes, engagement, or acceptability. However, in designing analyses to examine whether modifications may lead to differences in outcomes, it is important not to confound the impact of potential moderating factors that inspired the modification (e.g., comorbidity) with the impact of the modification itself.

We developed and reviewed some study methodologies, summarized elsewhere [1, 15], that can facilitate prospective research on modifications. We also reviewed experimental, prospective investigations of adaptations [1], but many of the adaptations and combinations thereof that occur in settings in which interventions routinely occur have not been represented. Methods to tease apart the impact of individual modifications when they frequently occur in conjunction with multiple others remain to be developed and may require large samples. Chambers and Norton [60] suggest the development of a database in which data from multiple projects can be pooled, using a common taxonomy, to facilitate more rapid understanding of what adaptations are necessary or effective for similar interventions when delivered to different populations or in different contexts. Within efforts to implement or scale-up across multiple sites, strategies such as qualitative comparative analysis may identify combinations of contextual factors and adaptations that associated with outcomes.

## Conclusion

Much work remains to be done to develop generalized knowledge about the process, nature, and outcomes of modifications made to different types of interventions in vastly different contexts. The FRAME is intended to capture information that reflects the complex and dynamic settings in which implementation occurs. Documenting with the FRAME can facilitate more rigorous study that includes efforts not only to characterize adaptations themselves, but also to clarify the timing, context, and process of modifying interventions to facilitate their implementation, scale-up, spread, and sustainment.

## Abbreviations

EBP: Evidence-based program/practice
FRAME: Framework for Reporting Adaptations and Modifications-Enhanced
MAC: Modification and Adaptation Checklist
NIH: National Institute of Health
QCA: Qualitative Comparative Analysis
RE-AIM: Reach, Effectiveness, Adoption, Implementation and Maintenance
